# Neoadjuvant Dovitinib in Early- and Intermediate-Stage Hepatocellular Carcinoma

**DOI:** 10.1093/oncolo/oyac168

**Published:** 2022-08-22

**Authors:** Alessandro Rizzo, Angela Dalia Ricci, Giovanni Brandi

**Affiliations:** Department of Experimental, Diagnostic and Specialty Medicine, S. Orsola-Malpighi University Hospital, Bologna, Italy; Department of Experimental, Diagnostic and Specialty Medicine, S. Orsola-Malpighi University Hospital, Bologna, Italy; Department of Experimental, Diagnostic and Specialty Medicine, S. Orsola-Malpighi University Hospital, Bologna, Italy

## Abstract

This letter to the editor comments on recently reported results of a phase II study of dovitinib therapy for hepatocellular carcinoma

We read with interest the article published by Woei-A-Jin et al and commend the authors for bringing this interesting report to the field of neoadjuvant systemic treatment in early- and intermediate-stage hepatocellular carcinoma (HCC).^1^ Currently, there is an urgent need for active systemic treatment as a preoperative strategy to achieve disease down-staging and expand the proportion of patients who may derive benefit from locoregional therapies.^2^ Despite optimal local control, most of patients with HCC recur, and the 5-year survival rate for early- and intermediate-stage HCC ranges between 15% and 50%, with failure to control micro-metastatic disease severely influencing early relapse in this setting.^3^

In *The Oncologist*, Woei-A-Jin et al reported the results of a phase II trial including 24 patients with early- and intermediate-stage HCC, treated with neoadjuvant oral dovitinib for 4 weeks, followed by locoregional therapy.^1^ Of note, the 48% of patients had an overall response, including 13% complete remission; however, tolerability has represented an important issue, with frequent dose reductions and interruptions. We believe some elements deserve discussion.

Firstly, although this pilot trial revealed important pharmacodynamic information, the sample size was small, and the rate of clinical benefit needs to be confirmed in a larger cohort. Secondly, from a safety point of view, conditions such as underlying cirrhosis and hepatotropic viral infection make the interaction between dovitinib and locoregional therapies deserving an accurate and comprehensive safety evaluation. In addition, there is currently a lack of efficacy data to confirm the activity of tyrosine kinase inhibitors (TKIs) in early-stage patients with HCC. In fact, the appropriate population in which neoadjuvant TKIs followed by locoregional therapies could be more beneficial still needs to be defined, and the trial included a widely varied patient population ranging from BCLC 0 to BCLC B, with the latter representing 45.8% of all subjects. In our view, these wide inclusion criteria are prone to dilute the effect of dovitinib and could have introduced some bias.

However, we believe that the authors are to be commended for their efforts. In fact, despite caution should always be exercised when interpreting results from single-arm, phase II trials conducted in a single center, the results of the study published by Woei-A-Jin et al are of great interest.^1^

Evaluation of the bioactivity of TKIs alone or in combination with other anticancer agents in early- and intermediate-stage HCC, where the biology of the disease significantly differs from that of advanced HCC, remains an important research aim.^4,5^ Future clinical trials specifically focused on which patients could benefit more from these treatments will be important to improve clinical outcomes and to understand if TKIs followed by locoregional therapies could represent a new frontier for early- and intermediate-stage patients with HCC.

## References

[1] Woei-A-Jin FJSH , Weijl NI, et al. Neoadjuvant treatment with angiogenesis-inhibitor dovitinib prior to local therapy in hepatocellular carcinoma: a phase II study. The Oncologist. 2021;26(10):854-864. 10.1002/onco.13901 34251745 PMC8488766

[2] Llovet JM , Kelley RK, Villanueva A, et al. Hepatocellular carcinoma. Nat Rev Dis Primers. 2021;7(1):6. 10.1038/s41572-020-00240-3. 33479224 PMC13537433

[3] Llovet JM , Castet F, Heikenwalder M, et al. Immunotherapies for hepatocellular carcinoma. Nat Rev Clin Oncol. 2021. 10.1038/s41571-021-00573-2. 34764464

[4] Dikilitas M. Why adjuvant and neoadjuvant therapy failed in HCC. Can the new immunotherapy be expected to be better? J Gastrointest Cancer. 2020;51(4):1193-1196. 10.1007/s12029-020-00497-7. 32869146

[5] Pinato DJ , Cortellini A, Sukumaran A, et al. PRIME-HCC: phase Ib study of neoadjuvant ipilimumab and nivolumab prior to liver resection for hepatocellular carcinoma. BMC Cancer 2021;21(1):301. 10.1186/s12885-021-08033-x. 33757459 PMC7988931

